# Supplementary material for: Chromothripsis during telomere crisis is independent of NHEJ, and consistent with a replicative origin
Source: Genome Res. 2019 May;29(5):737–49. doi: 10.1101/gr.240705.118 (PMC6499312; doi:10.1101/gr.240705.118)
Supplement: Supplemental Material [file supp_gr.240705.118_Supplemental_file_1.zip › contigs/annotated_contigs/DB85/contig.2.DB85_length_771_mean_cov_6.42023346304.docx]

**DB85_length_771_mean_cov_6.42023346304**

```
TGTGACTGCTGGTGTTTTAGTAAGACAGAAGGGTACAGTGTTTACCCTGTACATGGTCAAGTGCAATGGTCAGGACACGTAGGGATGTT
 >chr12:120191906-120192217 - E=3e-172
GCAGGAATACAGCAACATGTTACATGGACTCTTGAGCCAGACTTTCTGGGTTTGAATGCCAGTCCTGCCTTTTAAAAAGATTTTTATAG

CTGTGTGACATTTGCTCAGTTACCTAATCTCTCAGTGCCACAGTTTGCTTATCTGTAAAATGGAGGTAATAGTAGTACCTCCCTTAGTG

AGTTGTTCTGAAAATGAAATGAGTTAATTCAGGGCCCAGTGTG|T|GATCTTTAGGACCACACTGTCCATGTTTGTCAGGTCAATAGAT
                                            >chr12:120190491-120190952 - E=2e-256
GTGTCGTGGTCTCCCTCACCCCCATACCTTTGGATGTCTTGCTTCCCCTGCCTGGAATTTTCTTTCACCTCTTACCTGCTTGTTTGACT

CCTGCATATTCTTCAACAGAATTTAACAGATCCAGAGTTAGCTCCTCTACCCACCTGCGGTATTCCTGCAACATCCCTATATGTCCTGA

CCGTTGCACTTGACCATGATATATGGTCCTGGTTGGTTTTCATTTTTCTCCCCACTTGAAGGCAGAGACTGTCTTTCATCTCCATATTC

CATTTTCCACAGAAATTGCTGAGTAAATTTTTGTTAAATGAATGATGAAAGATTGAATAGATAGGTGGATGGATGAGCAGATGGAGGGA

AGAAGGATTAACTTCTAATGTGACATTAAAGCAGATTACCTAATGAGGACACATCAGAGAA
```
